# 219. Clinical Outcomes of Early vs Late Oral Stepdown Therapy in the Management of Uncomplicated Gram-Negative Bacteremia

**DOI:** 10.1093/ofid/ofad500.292

**Published:** 2023-11-27

**Authors:** Amanda Michael, Nikunj M Vyas, Joseph DeRose

**Affiliations:** Jefferson Health New Jersey, Tabernacle, New Jersey; Jefferson Health New Jersey, Tabernacle, New Jersey; Jefferson Health New Jersey, Tabernacle, New Jersey

## Abstract

**Background:**

Current studies show that oral stepdown antibiotics are safe and effective for patients with gram-negative bacteremia (GNB), but evidence is lacking in appropriate time from IV to oral stepdown therapy. The purpose of this study was to evaluate clinical outcomes in early (EPOS) vs late (LPOS) oral stepdown therapy in patients with GNB.

**Methods:**

This was an IRB-approved retrospective chart review including patients in a three hospital community health system. Patients were included in this study if they were ≥ 18 years old, hospitalized for ≥ 24 hours with a positive blood culture with *E. coli, Klebsiella spp*., and/or *Proteus spp.* and transitioned to oral antibiotics prior to discharge. Exclusion criteria included complicated bacteremia and any patients who were pregnant or immunocompromised. The primary endpoint of the study was to evaluate the achievement of clinical cure at the end of therapy (CC) in EPOS (≤ 96 hours) vs LPOS ( > 96 hours) groups. A subgroup analysis included achievement of microbiological cure (MC). The secondary endpoint evaluated the length of stay (LOS) and duration of IV (DIV) and duration of PO (DPO) antibiotics.

**Results:**

Total of 50 patients were included in the primary analysis, with 30 patients in the EPOS group and 20 patients in the LPOS group. The median age and gender stratification between both groups were similar. CC (93.3% vs 100%) and MC (86.7% vs 95%) were similar amongst patients in the EPOS and LPOS groups, respectively. As seen in Table 1, DIV was shorter in the EPOS group (3.27 vs 6.2 days, P=0.0001) and DPO was longer in the EPOS (6.83 vs 4.75 days, P=0.007). Patients in the EPOS group had trends toward shorter LOS compared to the LPOS group (6.67 vs 8.1 days, P=0.08). Patient is the EPOS group were more likely to receive oral cephalosporins compared to LPOS patients (83.3% vs 45%, P=0.006) as seen in figure 1, patients in the LPOS group were more likely to receive amoxicillin/clavulanate than the EPOS patients (30% vs 6.7%, P=0.04).

**Figure:**
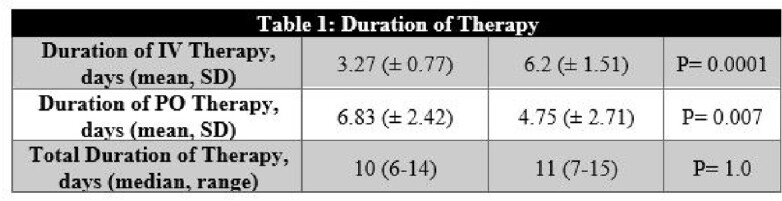

**Figure:**
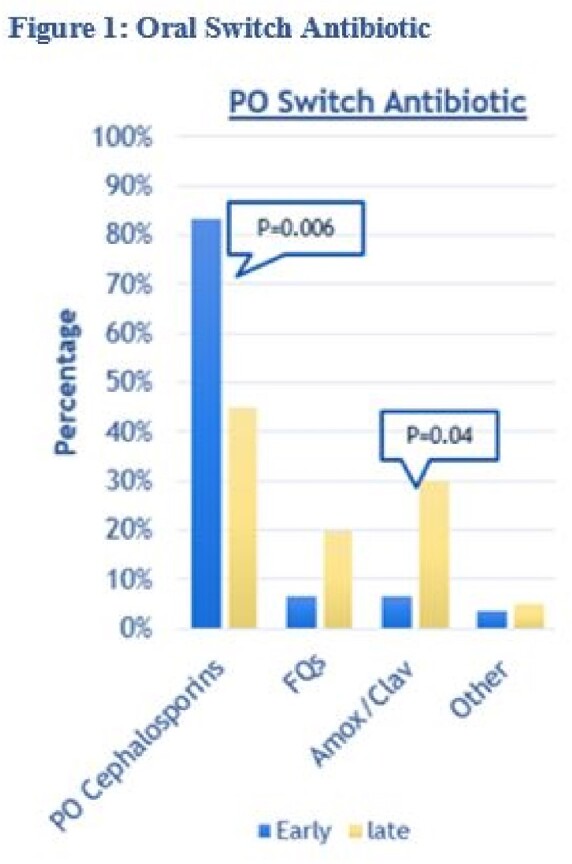

**Conclusion:**

EPOS may be a viable approach to uncomplicated gram-negative bacteremia with a lot of practical benefits. EPOS may lead to decreased DIV therapy and potentially decreased LOS. Additionally, EPOS with cephalosporins may be considered in most patients with uncomplicated bacteremia with a urinary source.

**Disclosures:**

**All Authors**: No reported disclosures

